# Endoscopic techniques to reduce recurrence rates after colorectal EMR: systematic review and meta-analysis

**DOI:** 10.1007/s00464-021-08574-z

**Published:** 2021-06-02

**Authors:** Gijs Kemper, Ayla S. Turan, Erik J. Schoon, Ruud W. M. Schrauwen, Ludger S. M. Epping, Christian Gerges, Torsten Beyna, Horst Neuhaus, Ufuk Gündug, Peter D. Siersema, Erwin J. M. van Geenen, the ENDOCARE Study Group

**Affiliations:** 1 Department of Gastroenterology and Hepatology, Radboud University Medical Center, Radboud Institute for Health Sciences, 6500 HB Nijmegen, The Netherlands; 2 Department of Gastroenterology and Hepatology, Catharina Hospital Eindhoven, Eindhoven, The Netherlands; 3 Department of Gastroenterology and Hepatology, Bernhoven, Uden, The Netherlands; 4 Department of Gastroenterology and Hepatology, Maasziekenhuis Pantein, Boxmeer, The Netherlands; 5 Department of General Internal Medicine and Gastroenterology, Evangelical Hospital Düsseldorf, Düsseldorf, Germany; 6 Department of Internal Medicine and Gastroenterology, Katholisches Karl Leisner Klinikum - St.-Antonius-Hospital Kleve, Kleve, Germany

**Keywords:** Endoscopic mucosal resection, Local neoplasm recurrence, Colonic polyps

## Abstract

**Background:**

Colorectal endoscopic mucosal resection (EMR) is an effective, safe, and minimally invasive treatment for large lateral spreading and sessile polyps. The reported high recurrence rate of approximately 20% is however one of the major drawbacks. Several endoscopic interventions have been suggested to reduce recurrence rates. We conducted a systematic review and meta-analysis to assess the efficacy of endoscopic interventions targeting the EMR margin to reduce recurrence rates.

**Methods:**

We searched in PubMed and Ovid for studies comparing recurrence rates after interventions targeting the EMR margin with standard EMR. The primary outcome was the recurrence rate at the first surveillance colonoscopy (SC1) assessed histologically or macroscopically. For the meta-analysis, risk ratios (RRs) were calculated and pooled using a random effects model. The secondary outcome was post-procedural complication rates.

**Results:**

Six studies with a total of 1335 lesions were included in the meta-analysis. The techniques performed in the intervention group targeting the resection margin were argon plasma coagulation, snare tip soft coagulation, extended EMR, and precutting EMR. The interventions reduced the adenoma recurrence rate with more than 50%, resulting in a pooled RR of 0.37 (95% CI 0.18, 0.76) comparing the intervention group with the control groups. Overall post-procedural complication rates did not increase significantly in the intervention arm (RR 1.30; 95% CI 0.65, 2.58).

**Conclusion:**

Interventions targeting the EMR margin decrease recurrence rates and may not result in more complications.

**Supplementary Information:**

The online version contains supplementary material available at 10.1007/s00464-021-08574-z.

Colorectal cancer (CRC) is worldwide the third and second most common cancer in men and women, respectively [1]. CRC predominantly develops from premalignant polypoid lesions of the colon. Colonoscopy with polypectomy is able to detect and subsequently remove these (pre)malignant lesions, resulting in a decreased mortality rate from CRC [2]. Therefore, CRC screening programs are worldwide implemented to detect and remove colonic polyps and/or cancer at an early stage [3].

Endoscopic resection of small polyps is a straightforward routine procedure. However, the resection of larger lateral spreading lesions (LSL) and sessile polyps requires more advanced endoscopic techniques [4]. Nowadays the standard treatment for larger lesions is endoscopic mucosal resection (EMR) and in even more advanced lesions endoscopic submucosal dissection (ESD) [5, 6]. Colonic EMR is an effective, safe, and minimally invasive outpatient therapy. One of the greatest drawback is the high polyp (adenoma) recurrence rate of up to approximately 20% [7, 8]. Several risk factors for adenoma recurrence have been reported such as piecemeal resection, intraprocedural bleeding, high-grade dysplasia, and lesion size ≥ 40 mm [9].

Adenoma recurrences are often treated with re-EMR, avulsion techniques, ESD, full thickness resection, and in some cases surgical resection. These recurrences lead to intensified surveillance programs, increasing the burden for patients and healthcare systems in terms of quality of life and costs. Several methods have been suggested to reduce polyp recurrence, i.e., cauterization of the polyp resection margin with argon plasma coagulation (APC) [10] or snare tip soft coagulation (STSC) [11], enhancing optical imaging using underwater EMR [12] and additional circumferential removal of normal mucosa adjacent to the resection margins with a flex knife: precutting EMR [13]. Due to the lack of large randomized controlled trials (RCTs), these techniques are not yet incorporated in clinical guidelines and their application varies widely depending on the endoscopist’s preferences.

This systematic review and meta-analysis aim to assess the effect of endoscopic interventions targeting the EMR resection margin in order to reduce recurrence rates in large lateral spreading lesions and sessile polyps.

## Material and methods

### Search strategy

We conducted a systematic literature search in the electronic databases MEDLINE (PubMed) and EMBASE (Ovid) on February 4, 2020. We adhered to the Preferred Reporting Items for Systematic Reviews and Meta-Analyses (PRISMA) guidelines, by using a predefined protocol to identify studies reporting on colorectal lesions, endoscopic mucosal resection, and locoregional recurrence [14]. An experienced medical librarian assisted with the literature search. For both databases, we used search terms including colorectal lesions, endoscopic mucosal resection, and locoregional recurrence in article title and abstract. Because of the variety of possible techniques, the type of endoscopic intervention was not specified. Details of the search strategy can be found in the supplement. Since this study analyzes datasets, ethical approval was not required.

### Inclusion and exclusion criteria

We included studies in human subjects published in the English language in peer-reviewed journals addressing EMR recurrence rates and applying endoscopic interventions focusing on the EMR margin in lateral spreading lesions and sessile polyps > 15 mm. Inclusion criteria were RCTs or observational studies including a control group. Recurrence rates had to be reported at the first surveillance colonoscopy (SC1). Exclusion criteria were studies without original data or full-text, studies on endoscopic removal of malignant polyps, studies conducted in a pediatric population (< 18 years), and studies with a sample size < 10 lesions.

### Study selection

Eligibility was independently assessed by two authors (GK and AT) by screening title and abstract and subsequently including articles after evaluating the full text. Discrepancies between the two reviewers were resolved after discussion with a third reviewer (EvG).

### Outcomes

The primary outcome was the recurrence rate at SC1 for additional endoscopic interventions targeting the resection margin compared to the conservative approach with standard EMR. Although histopathology is the golden standard for recurrence assessment, most included articles evaluated recurrence endoscopically since endoscopic detection of recurrent adenoma is supposed to be highly accurate [15]. We therefore collected the endoscopic recurrence rate when histopathology evaluation was not available. Post-procedural complication rates were also collected as a secondary outcome including delayed bleeding, post-polypectomy (electrocoagulation) syndrome, and perforation.

### Risk of bias assessment

Risk of bias was assessed for each study outcome. For RCTs, we assessed the risk for bias using the Revised Cochrane risk-of-bias tool for randomized trials (RoB 2) [16] and the Methodological Index for Non-Randomized Studies (MINORS) checklist [17] for observational studies. The outcomes within each RCT were considered to contain low risk of bias, high risk of bias, or some concerns following the RoB 2 algorithm. The MINORS checklist for observational studies provides a total score on a scale of 0 to 24. In this review, a score of 0–8 was defined as high risk for bias, 9–16 was defined as some concerns, and a score of 17–24 was considered to represent a low risk for bias. In order to detect potential publication bias, a funnel plot was drawn to evaluate possible asymmetry.

### Statistical analysis

For the meta-analysis, we calculated risk ratios (RRs) for adenoma recurrence comparing the intervention with standard EMR. The RRs from the individual studies were pooled in this meta-analysis using the DerSimonian and Laird method with a random effects model. Heterogeneity among studies was assessed using the *I*^2^ (inconsistency) statistic. Values of 25%, 50%, and 75% indicate low, moderate, and high levels of heterogeneity, respectively [18]. Sensitivity analysis was performed containing only RCT data and excluding the observational studies. In order to evaluate the effect of the cauterization techniques and the techniques removing additional mucosa separately, subgroup analyses were conducted with the APC/STSC studies and the extended EMR/precutting studies, respectively. In addition, post-procedural complication rates were calculated from all performed EMRs in the intervention and in the standard EMR groups, respectively, for each study. Complication rates of the two groups were compared with a chi-square test. Subgroup analyses were performed pooling the complication rates for the cauterization techniques and for the intervention removing additional mucosa. *P-*values of < 0.05 were considered significant for all analysis. The analyses were conducted using Review manager version 5.3.5 and IBM SPSS Statistics version 25.

### Certainty of evidence

The certainty of evidence per outcome was evaluated with the Grading of Recommendations Assessment, Development and Evaluation (GRADE) approach [19].

## Results

### Study selection

**Fig. 1:**
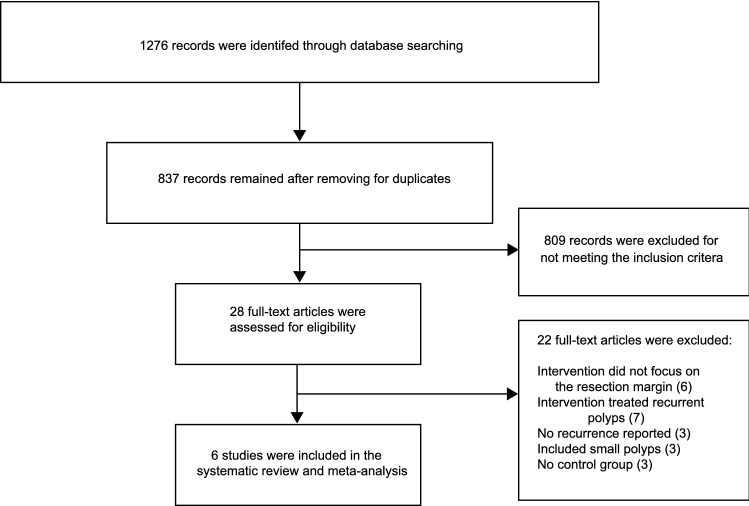
Flow diagram of the selection process

The electronic database search on MEDLINE and EMBASE identified 1276 records of which 837 remained after removing duplicates (Fig. 1). A total of 809 records were excluded for clearly not meeting the inclusion criteria. 28 articles underwent full-text examination after which another 22 were excluded prior to inclusion. Reasons for exclusion were that the intervention did not focus on resection margins (*n* = 6), the intervention was only used to treat recurrences (*n* = 7), the article did not report recurrence rates (*n* = 3), the studies also included lesions < 15 mm (*n* = 3), and studies did not have a control group (*n* = 3).

### Study characteristics

A total of 6 studies were selected for the meta-analysis which all evaluated endoscopic interventions in the colorectum targeting the EMR margin and reported recurrence rates at SC1. Three were randomized controlled trials including one multicenter trial. In the three observational studies, data were prospectively collected in one study, while in the other two data were retrospectively collected. The examined interventions were APC, STSC, extended EMR, and precutting EMR. Surveillance intervals between EMR and SC1 ranged between 3 and 12 months.

### Risk of bias

**Fig. 2:**
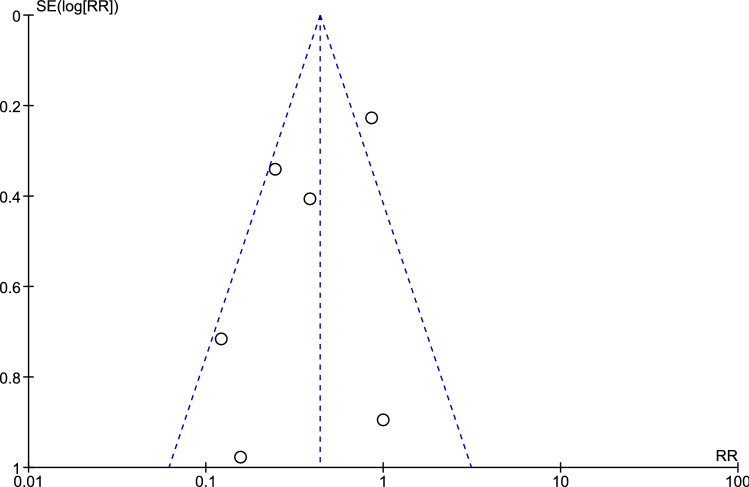
Funnel plot of the included studies. *RR* risk ratio, *SE* standard error

Table 1 shows the baseline study characteristics including the risk of bias for each outcome. Due to study design, the inability to blind endoscopic personnel during the procedure caused the greatest issue in preventing bias in the RCTs. Another potential source of bias for recurrence was detection bias. Detection bias is the result of assessing polyp recurrence endoscopically. Endoscopic records and the EMR resection site features provide the endoscopist information about the allocated intervention. In accordance with the RCTs, the assessment of polyp recurrence was a major issue in potentially causing detection bias since the assessment was performed endoscopically and not in all cases histopathologically. Supplementary Tables 2A–C and 3A–C show the risk of bias of the included studies for each domain. Figure 2 shows a funnel plot assessing publication bias. The plot appears to be symmetric suggesting that no publication bias was present, although we were unable to confirm this observation objectively due to a small number of studies making additional statistical analyses inappropriate [20].

**Table 1:** Baseline characteristics of the included studies

Study	Design	Intervention	No. of lesions intervention group	No. of lesions control group	Risk of bias for each outcome	
					Recurrence	Post-procedural complications
Albuquerque (2013) [28]	RCT	APC	10	10	Low	Low
Brooker (2002) [10]	RCT	APC	10	11	Low	Low
Kandel (2019) [23]	Retro	STSC	60	60	Low	Some concerns
Klein (2019) [11]	RCT	STSC	192	176	Some concerns	Low
Bahin (2016) [13]	Pro	Extended EMR	296	333	Low	Low
Lee (2012) [27]	Retro	Precutting EMR	64	113	Some concerns	Low

*RCT* randomized controlled trial, *Retro* retrospective, *Pro* prospective, *APC* argon plasma coagulation, *STSC* snare tip soft coagulation, *EMR* endoscopic mucosal resection

### Meta-analysis

**Fig. 3:**
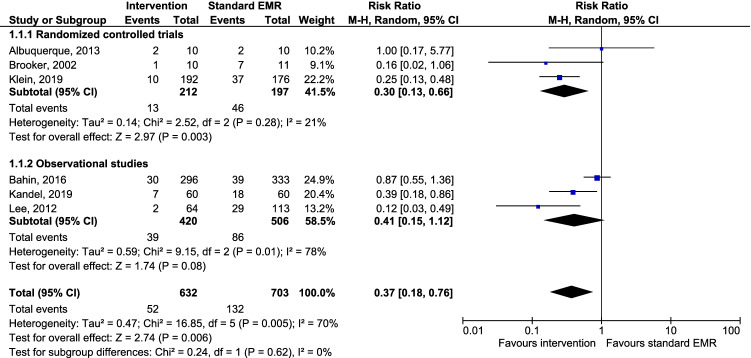
Forest plot of the pooled and individual risk ratios for recurrence in the intervention groups versus groups receiving standard EMR. *EMR* endoscopic mucosal resection, *CI* confidence interval

A total of 1335 lesions were assessed for recurrence at SC1 and included in the meta-analysis. The overall recurrence rate was 8.2% in the intervention groups versus 18.8% in the control groups. The pooled risk ratio for adenoma recurrence in the intervention groups was 0.37 (95% CI 0.18, 0.76) compared to the control groups. Figure 3 presents the pooled and individual risk ratios for each study. Heterogeneity between the included studies was assessed using *I*^2^ = 70%, which is considered to be moderate/high–high, although the power of this test in meta-analyses with small numbers of studies is low [21]. *I*^2^ dropped to 21% with a pooled RR of 0.30 (95% CI 0.13, 0.66) when only RCTs were included for the sensitivity analysis. The subgroup analysis comparing APC and STSC as cauterization techniques with the control groups showed a pooled RR of 0.31 (95% CI 0.19, 0.50; *I*^2^ = 0%). Including the precutting EMR and extended EMR studies, representing technique for additional mucosa removal only, resulted in a non-significant pooled RR of 0.36 (95% CI 0.05, 2.70; *I*^2^ = 87%). The certainty of evidence for the RR of recurrences was considered high for the RCTs and moderate for observational studies (Table 3).

### Complications

All included studies reported complication rates for the intervention and standard EMR groups. Table 2 shows the absence of statistical difference in delayed bleeding, post-polypectomy syndrome, or perforation rates in the intervention group versus the control group.

**Table 2:** Post-procedural complication rates

Study	Study group	DB rate in %	*p* value	PPS rate in %	*p* value	Perforation rate in %	*p* value
Albuquerque (2013) [28]	APC	0	NA	0	NA	0	NA
	Standard EMR	0		0		0	
Brooker (2002) [10]	APC	0	NA	0	NA	0	NA
	Standard EMR	0		0		0	
Kandel (2019) [23]	STSC	2	0.8	0	NA	NR	NA
	Standard EMR	3		0		NR	
Klein (2019) [11]	STSC	6.2	0.9	NR	NA	0.5	0.3
	Standard EMR	5.8		NR		1.5	
Bahin (2016) [13]	Extended EMR	5.9	0.6	NR	NA	3.6	0.5
	Standard EMR	5.1		NR		2.8	
Lee (2012) [27]	Precutting EMR	3	0.3	10	0.2	3	0.3
	Standard EMR	0		5.7		0	

*APC* argon plasma coagulation, *STSC* snare tip soft coagulation, *EMR* endoscopic mucosal resection, *NA* not applicable, *NR* not reported, *DB* delayed bleeding, *PPS* post-polypectomy syndrome

**Fig. 4:**
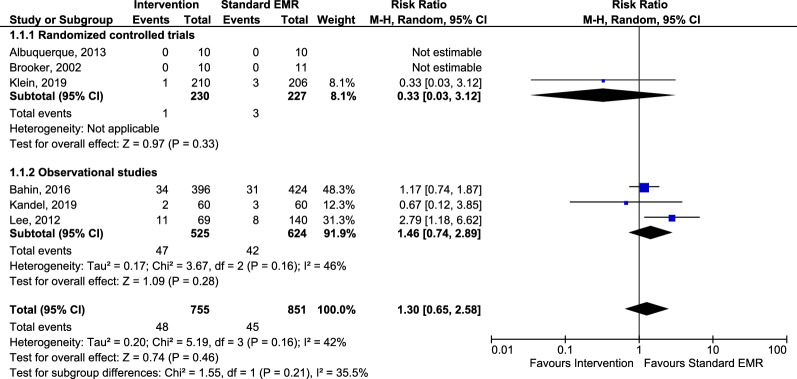
Forest plot of the pooled and individual risk ratios for post-procedural complications in the intervention groups versus groups receiving standard EMR. *EMR* endoscopic mucosal resection, *CI* confidence interval

Only Lee et al. reported a significant overall increase of post-procedural complications in the intervention group (precutting EMR) compared to the standard EMR group (15.9% versus 5.7%, *p* = 0.02) (Fig. 4). The pooled RR for post-procedural complications also did not show a significant result: 1.30 (95% CI 0.65, 2.58). In the subgroup analyses, risk ratios for pooled complication rates of the cauterization techniques versus standard EMR and the additional resection techniques versus standard EMR were 0.51 (95% CI 0.13, 2.03) and 1.67 (95% CI 0.73, 3.85), respectively. Table 3 shows the certainty of evidence of the post-procedural complication risk ratios for the RCTs (low) and the observational studies (moderate).

**Table 3:** GRADE approach to evaluate certainty of evidence

Outcomes	Risk of bias	Inconsistency	Indirectness	Imprecision	Publication bias	RR (95% CI)	Certainty
Recurrence 3 RCTs	Low	No	No	No	No	0.30 (0.13, 0.66)	High
Recurrence 3 observational studies	Low	Severe	No	No	No	0.41 (0.15, 1.12)	Moderate
Complications * 3 RCTs	Low	No	No	Very severe	No	0.33 (0.03, 3.12)	Low
Complications * 3 observational studies	Low	No	No	Severe	No	1.46 (0.74, 2.89)	Moderate

*RR* risk ratio

^*^Post-procedural complications

## Discussion

This meta-analysis demonstrates that interventions targeting the EMR margins result in a decrease in recurrence rates. Patients in whom the EMR margin is treated, are 63% less likely to have adenoma recurrence. No differences in adverse events potentially attributable to endoscopic treatment of the EMR margins were observed, suggesting that these treatments are safe.

This observation supports the hypothesis that the resection margin often still contains endoscopically invisible residual adenoma causing recurrences that can be treated with techniques that eliminate this residual tissue by cauterization or by additional circumferential resection. Subgroup analyses suggest that APC and STSC are potentially more efficient in preventing recurrence than the extended and the precutting EMR although these methods were not compared head to head in the meta-analysis.

Four of the six studies in our meta-analysis assessed APC and STSC as cauterization techniques for treating potential recurrent tissue. In order to reduce recurrence rates, APC is applied to the entire margin of the resection site leaving a white touch indicating the coagulated tissue. In line with our findings, a large retrospective multicenter cohort study from 2019 treating 264 lesions with APC resulted in a recurrence rate of only 4.5% [22]. The two studies in our meta-analysis that compared STSC with standard EMR reported similar results. The recurrence rate dropped from 21 and 30% in the control group to 5.2 and 12% in the STSC group, respectively [11, 23]. These results are promising, but the RCT of Klein et al. reports that adjuvant techniques to remove small polyp remnants after EMR were not allowed. This resulted in a non-radical resections rate of 10% and therefore the generalizability of this trial is reduced.

In order to assess which technique (APC versus STSC) is more successful in reducing the recurrence rate, a retrospective comparative study in 2019 assessed these two methods of thermal ablation on 101 lesions. It reported a non-statistical difference in recurrence rate of 10% for the APC group versus 7.8% in the STSC group [24].

Extended EMR and precutting EMR involve a more extensive resection with expanding the excision margin with at least 5 mm of normal appearing mucosa [13]. The recurrence rate in the extended EMR group was 10.1% versus 11.7% in the control group, which was not statistically significant. In this individual study, extending the EMR margins did not reduce the recurrence rate. A possible explanation could be that extending the EMR site was not always achieved, when visibility was compromised as a result of piecemeal cautery artifacts [25]. Another possible explanation for the non-superiority of extended EMR versus standard EMR may be the extensive use of STSC. Bahin et al. reported that minor residuals were treated with STSC in both groups. STSC was performed more often in the standard EMR group than in the extended EMR group (21.2% versus 14.3%, *p* = 0.012). Lee et al. compared precutting the mucosa circumferentially using a dual knife (precutting EMR) with standard EMR and reported a recurrence rate of 3.1% versus 25.7% (*p* = 0.001). A cohort study by Hong et al. without a control group reported no recurrences in 79 lesions treated with a similar technique. Low recurrence rates are potentially the result of achieving higher *en bloc* rates. Unlike the other techniques, precutting (or circumferential EMR) is however associated with an increased complication rate and requires additional training [26, 27].

This systematic review and meta-analysis comes with some limitations. First, it included studies with small sample sizes [10, 28], which increases the risk of a type 2 error [29]. Second, several studies were likely to have detection bias since recurrence was assessed endoscopically [11, 23, 27]. Although endoscopic assessment is proven to be accurate in evaluating recurrence [15], endoscopists could be biased considering they were aware of the allocated treatment. Third, the current number of studies available for this review was limited. Fourth, the level of heterogeneity in this meta-analysis is substantial. This is likely to be the result of the diversity of the performed interventions and study design, since the *I*^2^ reduced to 0% in the subgroup analysis. Nonetheless, we were unable to control for an uneven distribution of possible confounders due to missing data. Reported confounders include lesion size, piecemeal EMRs, and intraprocedural bleeding [9]. Fifth, one study excluded lesions which were macroscopically non-radical without additional treatment (cold avulsion and/or STSC) [11]. Sixth, most of the EMRs were performed in tertiary centers, which lowers the generalizability of the study in the general endoscopy practice. And last, alternative strategies not targeting the resection margin were not included in this meta-analysis. These techniques are based on enhancing optical imaging during the EMR and therefore theoretically reduce adenoma recurrence rates, such as underwater EMR [12, 30], cap-assisted EMR [22, 31], and wide-field EMR [32].

In order to overcome the limitations mentioned in this systematic review, we suggest the following design for future RCTs assessing techniques for reducing recurrence. First, we recommend a standardized definition of adenoma recurrence with histological confirmation in all EMRs. Next, optimization of generalizability can be achieved by including multiple non-tertiary centers and allowing additional treatment (such as STSC or avulsion techniques) for obtaining a complete resection. Details of the polyps (size, location, piecemeal resection rates, quality HD images) should be collected. Finally, a cost-effectiveness analysis should be included.

## Disclosures

This meta-analysis and systematic review was performed under the auspices of the ENDOCARE study group and subsidized by the European Union (INTERREG), Province of Gelderland and the Ministerium für Wirtschaft, Innovation, Digitalisierung und Energie des Landes Nordrhein-Westfalen. A commercial entity was not involved in the design, performance, and writing of the study. Peter D. Siersema receives unrestricted grants from Pentax (Japan), Norgine (UK), Motus GI (USA), MicroTech (China), and The eNose Company (Netherlands) and is in the advisory board of Motus GI (USA) and Boston Scientific (USA). Gijs Kemper, Ayla S. Turan, Eric J. Schoon, Ruud W.M. Schrauwen, Ludger S.M. Epping, Christian Gerges, Torsten Beyna, Horst Neuhaus, Ufuk Gündug, and Erwin J.M. van Geenen declare no conflict of interest.

## Supplementary Information

Below is the link to the electronic supplementary material.

Electronic supplementary material 1 (PDF 141 kb)
