# Supplementary material for: Endoscopic techniques to reduce recurrence rates after colorectal EMR: systematic review and meta-analysis
Source: Surg Endosc. 2021 Jun 2;35(10):5422–9. doi: 10.1007/s00464-021-08574-z (PMC8437853; doi:10.1007/s00464-021-08574-z)
Supplement: Supplementary file 1 — Electronic supplementary material 1 (PDF 141 kb) [file 464_2021_8574_MOESM1_ESM.pdf]

Supplementary

Supplement 1. Search strategy PubMed

("Colorectal Neoplasms"[Mesh] OR Colon neoplasm*[tiab] OR colon adenoma*[tiab] OR colon tumor*[tiab] OR colon tumour*[tiab] OR colonic neoplasm*[tiab] OR colonic tumor*[tiab] OR colonic adenoma*[tiab] OR colonic tumour*[tiab] OR colorectal neoplasm*[tiab] OR colorectal tumor*[tiab] OR colorectal tumour*[tiab] OR colorectal adenoma*[tiab] OR rectum tumor*[tiab] OR rectum tumour*[tiab] OR rectum adenoma*[tiab] OR rectum neoplasm*[tiab] OR rectal tumor*[tiab] OR rectal tumour*[tiab] OR rectal adenoma*[tiab] OR rectal neoplasm*[tiab] OR Colon polyp*[tiab] OR Colonic polyp*[tiab] OR Colorectal polyp*[tiab] OR laterally spreading[tiab] OR Rectum polyp*[tiab] OR Rectal polyp*[tiab]) AND ("Endoscopic Mucosal Resection"[Mesh] OR Endoscopic Mucosal Resection*[tiab] OR EMR[tiab] OR Polypectom*[tiab]) OR endoscopic piecemeal resection[tiab]) AND ("Neoplasm Recurrence, Local"[Mesh] OR recurrence[tiab])

Supplement 2A-C. Quality assessment RTCs

Table 2A. RoB 2 Revised Cochrane risk-of-bias tool for randomized trials. *Albuquerque et al.*				
	**Recurrence**		**Post procedural complication**	
**Domain**	**Judgement**	**Rationale**	**Judgement**	**Rationale**
Randomization process	Low risk	"After mucosectomy, patients were randomly assigned to the two groups".	Low risk	"After mucosectomy, patients were randomly assigned to the two groups".
Deviations from intended interventions	Low risk	No deviations from intended interventions.	Low risk	No deviations from intended interventions.
Missing outcome data	Low risk	No lost to follow-up.	Low risk	No lost to follow-up.
Measurement of the outcome	Low risk	Recurrence was assessed histologically. We assume that the pathologists were unaware of the allocation.	Low risk	We assume that all post procedural complications were documented properly.
Selection of the reported result	Low risk	All specified outcomes were reported.	Low risk	All specified outcomes were reported.
Overall bias	Low risk		Low risk	

Table 2B. RoB 2: Revised Cochrane risk-of-bias tool for randomized trials. *Brooker et al.*				
	**Recurrence**		**Post procedural complication**	
**Domain**	**Judgement**	**Rationale**	**Judgement**	**Rationale**
Randomization process	Low risk	"When the endoscopist judged that snare excision was complete, randomization to APC or control (non-APC) was performed".	Low risk	"When the endoscopist judged that snare excision was complete, randomization to APC or control (non-APC) was performed".
Deviations from intended interventions	Low risk	No deviations from intended interventions.	Low risk	No deviations from intended interventions.
Missing outcome data	Low risk	No lost to follow-up.	Low risk	No lost to follow-up.
Measurement of the outcome	Low risk	Recurrence was assessed histologically. We assume that the pathologists were unaware of the allocation.	Low risk	We assume that all post procedural complications were documented properly.
Selection of the reported result	Low risk	All specified outcomes were reported.	Low risk	All specified outcomes were reported.
Overall bias	Low risk		Low risk	

Table 2C. RoB 2: Revised Cochrane risk-of-bias tool for randomized trials. *Klein et al.*				
	**Recurrence**		**Post procedural complication**	
**Domain**	**Judgement**	**Rationale**	**Judgement**	**Rationale**
Randomization process	Low risk	"A computer generated random number table created in blocks of 100 was used for the allocation sequence".	Low risk	"A computer generated random number table created in blocks of 100 was used for the allocation sequence".
Deviations from intended interventions	Low risk	No deviations from intended interventions.	Low risk	No deviations from intended interventions.
Missing outcome data	Low risk	It is unlikely that the 11.5% lost to follow-up rate affects the recurrence rate.	Low risk	It is unlikely that post procedural complications were not documented in the respective clinical center.
Measurement of the outcome	Some concerns	Endoscopists assessing recurrence during the follow-up endoscopy were aware of the allocated treatment.	Low risk	We assume that all post procedural complications were documented properly.
Selection of the reported result	Low risk	All specified outcomes were reported.	Low risk	All specified outcomes were reported.
Overall bias	Some concerns		Low risk	

Supplement 3A-C. Quality assessment observational studies

Table 3A: Methodological index for non-randomized studies (MINORS) assessment tool. Bahin et al.				
	**Recurrence**		**Post procedural complication**	
**Items**	**Score**	**Comments**	**Score**	**Comments**
1 A clearly stated aim	2	Aim clearly described in introduction.	2	Aim clearly described in introduction.
2 Inclusion of consecutive patients	2	Inclusion and exclusion criteria are clearly stated.	2	Inclusion and exclusion criteria are clearly stated.
3 Prospective collection of data	2	Analysis was performed post-hoc, data was collected prospectively. Study protocol was registered.	2	Analysis was performed post-hoc, data was collected prospectively. Study protocol was registered.
4 Endpoints appropriate to the aim of the study	2	Biopsy proven adenoma at first surveillance colonoscopy.	1	Post procedural complications were named but not defined.
5 Unbiased assessment of study endpoint	2	Recurrence is assessed histologically.	2	We assume that the complications were documented unbiased.
6 Follow-up period appropriate to the aim of the study	2	Follow-up period was defined until first surveillance colonoscopy at 4 months.	2	Follow-up period was defined until first surveillance colonoscopy at 4 months.
7 Loss to follow-up less than 5%	0	12% of all patients were lost to follow-up.	2	We assume that all post procedural complications were documented at the respective clinical centers.
8 Prospective calculation of study size	0	Not reported.	0	Not reported.
9 An adequate control group	2	Control group consists of patients receiving standard EMR.	2	Control group consists of patients receiving standard EMR.
10 Contemporary groups	1	The 2 groups are not managed during the same period.	1	The 2 groups are not managed during the same period.
11 Baseline equivalence of groups	1	Some possible important characteristics are not equally distributed between the two groups. Additional thermal ablation is used in 21% in de control group vs 14% in the intervention group.	1	Some possible important characteristics are not equally distributed between the two groups. Additional thermal ablation is used in 21% in de control group vs 14% in the intervention group.
12 Adequate statistical analyses	2		2	
TOTAL	18/24		19/24	

Table 3B: Methodological index for non-randomized studies (MINORS) assessment tool. Kandel et al.				
	**Recurrence**		**Post procedural complication**	
**Items**	**Score**	**Comments**	**Score**	**Comments**
1 A clearly stated aim	2	Clearly described in the introduction.	2	Clearly described in the introduction.
2 Inclusion of consecutive patients	2	Single center between 2016 and 2017.	2	Single center between 2016 and 2017.
3 Prospective collection of data	0	Retrospective study design.	0	Retrospective study design.
4 Endpoints appropriate to the aim of the study	2	Recurrence at first surveillance colonoscopy.	1	Post procedural complications were named but not defined.
5 Unbiased assessment of study endpoint	1	Biopsies were not taken if the endoscopist was confident there was no recurrence.	2	We assume that the complications were documented unbiased.
6 Follow-up period appropriate to the aim of the study	2	Follow-up period was defined until first surveillance colonoscopy at 6 months.	2	Follow-up period was defined until first surveillance colonoscopy at 6 months.
7 Loss to follow-up less than 5%	2	No patients were lost to follow-up.	2	No patients were lost to follow-up.
8 Prospective calculation of study size	2		0	Not reported.
9 An adequate control group	2	Control group consist of patients who received standard EMR procedure.	2	Control group consist of patients who received standard EMR procedure.
10 Contemporary groups	1	The control group consists of patients who were treated with standard EMR prior to the start of the intervention.	1	The control group consists of patients who were treated with standard EMR prior to the start of the intervention.
11 Baseline equivalence of groups	2	Baseline characteristics for the most important possible confounders show no differences between the two groups.	2	Baseline characteristics for the most important possible confounders show no differences between the two groups.
12 Adequate statistical analyses				
TOTAL	18/24		16/24	

Table 3C: Methodological index for non-randomized studies (MINORS) assessment tool. Kandel et al.				
	**Recurrence**		**Post procedural complication**	
**Items**	**Score**	**Comments**	**Score**	**Comments**
1 A clearly stated aim	2	Clearly described in the introduction.	2	Clearly described in the introduction.
2 Inclusion of consecutive patients	2	All patients between 2004 and 2009 were included.	2	All patients between 2004 and 2009 were included.
3 Prospective collection of data	0	Retrospective study design.	0	Retrospective study design.
4 Endpoints appropriate to the aim of the study	2	Recurrence at first surveillance colonoscopy.	1	Post procedural complications were named but not defined.
5 Unbiased assessment of study endpoint	1	Biopsies were only taken to confirm endoscopic visible recurrences.	2	We assume that the complications were documented unbiased.
6 Follow-up period appropriate to the aim of the study	2	6 to 12 months after the EMR.	2	6 to 12 months after the EMR.
7 Loss to follow-up less than 5%	0	32/209 patients were lost to follow up = 15%.	2	We assume that all post procedural complications were documented at the respective clinical centers.
8 Prospective calculation of study size	0	Not reported.	0	Not reported.
9 An adequate control group	2	Control group consist of patients who received standard EMR procedure.	2	Control group consist of patients who received standard EMR procedure.
10 Contemporary groups	1	The standard EMRs were performed between 2004 and 2009. Percutting-EMRs were performed between 2006 and 2009.	1	The standard EMRs were performed between 2004 and 2009. Percutting-EMRs were performed between 2006 and 2009.
11 Baseline equivalence of groups	2	Most important possible confounders are compared between the two groups. There are no significant differences.	2	Most important possible confounders are compared between the two groups. There are no significant differences.
12 Adequate statistical analyses	2		2	
TOTAL	16/24		18/24	
